# Myricetin Amplifies Glucose–Stimulated Insulin Secretion via the cAMP-PKA-Epac-2 Signaling Cascade

**DOI:** 10.3390/biomedicines13061447

**Published:** 2025-06-12

**Authors:** Akhtar Ali, Zahida Memon, Abdul Hameed, Zaheer Ul-Haq, Muneeb Ali, Rahman M. Hafizur

**Affiliations:** 1 Department of Pharmacology, Ziauddin University Karachi, Karachi 75000, Pakistan; akhtar.ali@zu.edu.pk (A.A.); zahida.memon@zu.edu.pk (Z.M.); 2 Ziauddin College of Molecular Medicine, Ziauddin University Karachi, Karachi 75000, Pakistan; 3 Department of Molecular Medicine, Ziauddin University Karachi, Karachi 75000, Pakistan; 4 Dr. Panjwani Center for Molecular Medicine and Drug Research, International Center for Chemical and Biological Sciences, University of Karachi, Karachi 75270, Pakistan; zaheer.qasmi@iccs.edu (Z.U.-H.); alimunib33@gmail.com (M.A.); hafizpcmd@yahoo.com (R.M.H.); 5 Department of Biochemistry and Molecular Biology, Dhaka International University, Dhaka 1212, Bangladesh; 6 Daffodil International University, Daffodil Smart City, Dhaka 1216, Bangladesh

**Keywords:** myricetin, insulin secretion, sulfonylurea, antidiabetic drugs, insulin secretagogues

## Abstract

**Aim**: Myricetin, a natural bioflavonoid, is reported as an anti-diabetic agent since it possesses the ability to inhibit α-glucosidase activity, stimulate insulin action and secretion, manage ROS, and prevent diabetes complications. Myricetin was identified as a new insulin secretagogue that enhances glucose-stimulated insulin secretion and seems like a better antidiabetic drug candidate. Here, we explored the insulinotropic mechanism(s) of myricetin *in vitro* in mice islets and *in silico*. **Methods**: Size-matched pancreatic islets were divided into groups and incubated in the presence or absence of myricetin and agonists/antagonists of major insulin signaling pathways. The secreted insulin was measured by ELISA. Molecular docking studies were performed with the key player of insulin secretory pathways. **Results**: Myricetin dose-dependently enhanced insulin secretion in isolated mice islets, and its insulinotropic effect was exerted at high glucose concentrations distinctly different from glibenclamide. Myricetin-induced insulin secretion was significantly inhibited using the diazoxide. Furthermore, myricetin amplified glucose-induced insulin secretion in depolarized and glibenclamide-treated islets. Myricetin showed an additive effect with forskolin- and IBMX-induced insulin secretion. Interestingly, H89, a PKA inhibitor, and MAY0132, an Epac-2 inhibitor, significantly inhibited myricetin-induced insulin secretion. The *in silico* molecular docking studies further validated these *in vitro* findings in isolated pancreatic islets. **Conclusions**: Myricetin, a potential natural insulin secretagogue, amplifies glucose-induced insulin secretion *via* the cAMP-PKA-Epac-2 signaling pathway.

## 1. Introduction

The pathophysiology of type 2 diabetes (T2D) involves the development of insulin resistance and pancreatic β-cell dysfunction, which occur due to the interaction of local abnormalities within different tissues and lead to systemic dysregulation of glucose metabolism [1]. Regarding the pathophysiology of diabetes, it has been documented that the Asian population has predominant insulin secretory impairments; however, insulin resistance is the major cause in the Western population. Therefore, the proposed treatment option for diabetic patients should be based on the pathophysiological requirements of the population [2]. Amongst the oral hypoglycemic drugs, sulfonylurea and the glinide group have been regarded as insulin secretagogues; however, due to hypoglycemia and other side effects, these drugs are not considered as the first choice of treatment for diabetes [3]. Additionally, continuous activation of K_ATP_ by marketed insulin secretagogues such as sulfonylurea increases stress on the β-cells, further leading to β-cell damage and worsening the condition [4]. Insulin secretion by the β cells is a biphasic process. In the first phase, higher concentrations of glucose stimulate K_ATP_ channels, leading to membrane depolarization. This facilitates the Ca^2+^ entry, which triggers an insulin secretory burst through the plasma membrane. However, the second phase involves the activation of intracellular amplifying pathways, which maintain sustained insulin release in response to glucose concentrations [5]. Our group took the initiative to investigate various natural compounds with amplification of insulin secretion at stimulated glucose concentrations and explore the exclusive glucose-dependent insulin secretory mechanism(s) [6,7].

**Figure 1:**
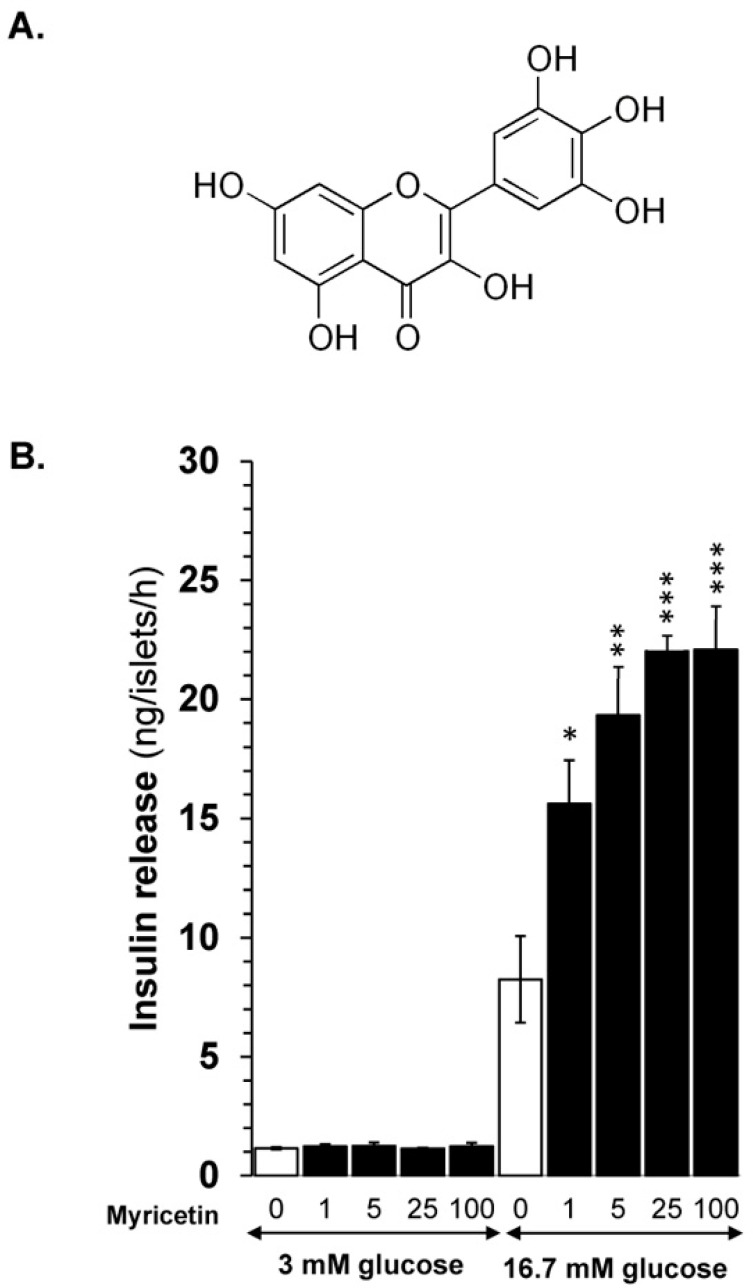
(**A**) Myricetin structure. (**B**) Myricetin enhances GSIS in isolated mice islets in a dose-dependent manner. Myricetin was added at 0, 1, 5, 25, and 100 μM concentrations in the presence of 3 mM or 16.7 mmol/L glucose. Myricetin does not induce insulin secretion at a 3 mM glucose concentration; however, at 16.7 mmol/L, a significant dose-dependent increase in insulin secretion was observed. * *p* < 0.05, ** *p* < 0.01, and *** *p* < 0.001 denote significant changes over the respective control values. The mean and SEM for the analysis were calculated based on five independent experiments conducted in triplicates.

Myricetin (Figure 1A), a natural flavonoid, has been isolated from many plant extracts, including *Myricaceae*, *Polygonaceae*, *Primulaceae*, *Pinaceae*, *Anacardiaceae*, and *Trigonella foenum-graecum* (Fenugreek) [8,9,10]. It has shown anticancer, anti-inflammatory, and antidiabetic properties in various *in vitro* and *in vivo* models [11]. The anti-diabetic properties of myricetin are attributed to its ability to regulate glucose by various mechanisms, including its association with glucose transporter-4, an increase in insulin sensitivity, and the inhibition of islet β cell apoptosis [12]. Furthermore, a recent study has highlighted that myricetin is a potent natural activator of the GLP-1 receptor [13]. Additionally, it has been reported that myricetin has the potential to secrete insulin at a stimulated glucose concentration [14].

According to previously reported data, myricetin has a direct effect on the GLP-1 receptor [13]. However, our preliminary results showed that myricetin exerts an additive effect in forskolin and IBMX-induced insulin secretion. These preliminary results suggest that, in addition to its effect on the GLP-1 receptor, myricetin has a strong amplifying effect on the downstream signaling pathways.

Based on the extensive use of myricetin as a broad pharmacological agent with a potential glucose-lowering effect and on our preliminary results with its effect on the amplifying pathway, the current study was designed to explore the mechanism(s) of myricetin on triggering and amplifying signaling cascades, which has not been documented in the literature yet.

## 2. Methodology

### 2.1. Materials

Collagenase V, bovine serum albumin (BSA), glibenclamide (GB), forskolin, 3- 3-isobutyl-1-methylxanthine (IBMX), diazoxide, U0126, H89, MAY 0132, verapamil, SQ22536, and 96% pure Myricetin were purchased from Sigma (St. Louis, MO, USA). Mouse insulin ELISA kits were purchased from Crystal Chem USA (Elk Grove Village, IL, USA).

### 2.2. Animals

Male BALB/c mice aged 6-8 weeks, weighing 30–40 g, were obtained from the animal house of the International Center for Chemical and Biological Sciences (ICCBS), University of Karachi, Pakistan. The animals were housed in conditions with controlled temperature and humidity (25 ± 2 °C and 50–55%, respectively) with a 12h light/dark cycle. The mice were kept in hygienic cages with unrestricted access to food and water. The study was approved by the Animal Ethics Committee of Ziauddin University, and protocol number 2022-07/AA/FHS was allotted.

### 2.3. Pancreatic Islet Isolation from Mice

The pancreatic islets were isolated from 3 to 5 mice for one set of experiments. To validate the experimental results, all the experiments were performed in triplicates. The pancreas of male BALB/c mice was distended by administering 3 mL of collagenase V solution (1 mg/kg) through the common bile duct after they were sedated with sodium thiopental (30 mg/kg). Subsequently, the pancreas was extracted and digested for 15 min at 37 °C in a collagenase mixture. The isolated islets were washed 2–3 times with Hank’s Balanced Salt Solution (HBSS) and hand-picked under a NIKON SMZ-745T stereomicroscope.

### 2.4. Glucose-Stimulated Insulin Secretion Assay

The isolated islets were incubated with 3 mM glucose for 45 min at 37 °C in Krebs-Ringer bicarbonate buffer (KRBB; 118 mM NaCl; 4.7 mM KCl; 1.9 mM CaCl2; 1.2 mM MgSO_4_; 1.2 mM KH_2_PO_4_; and 25 mM NaHCO_3_; 10 mM HEPES; and 0.1% BSA, pH 7.4). Following initial incubation, batches of size-matched islets (three islets in each batch) were incubated for 60 min at 37 °C in KRBB containing either 3 mM (basal) or 16.7 mM (stimulatory) glucose supplemented with/without myricetin [6,7].

### 2.5. Identification of Optimal Dose

The stock solution of myricetin was prepared using DMSO, which was further diluted to a working concentration. It was ensured that in the final working solution, the concentration of DMSO should be less than 0.25%. The different batches of mouse islets were treated with different concentrations of myricetin (i.e., 0, 1, 5, 25, and 100 μM) at 3 mM and 16.7 mM glucose concentrations for the identification of the optimal dose.

### 2.6. Identification of Mechanism of Action

After the optimal dose selection, myricetin was proceeded to the insulin secretory mechanism(s). For these experiments, batches of mice islets were incubated at 16.7 mM glucose with or without myricetin (25 μM) in the presence or absence of pharmacological agonists/antagonists, such as 16.7 mM glucose with 10 μM glibenclamide, with or without myricetin; 16.7 mM glucose with 50 μM diazoxide with or without myricetin; 16.7 mM glucose with 50 μM diazoxide + KCl (25 mM) with or without myricetin; 16.7 mM glucose with 200 μM verapamil with or without myricetin; 16.7 mM glucose with 100 μM isobutyl methylxanthine (IBMX), an inhibitor of phosphodiesterase with or without myricetin; 16.7 mM glucose with H-89 (50 μM), a PKA inhibitor, with or without myricetin; 16.7 mM glucose with U0126 (20 μM), a MEK kinase inhibitor with or without myricetin; 16.7 mM glucose with SQ22536 (25 μM), an adenylate cyclase inhibitor with or without myricetin. The mouse insulin ultra-sensitive ELISA kit was used for insulin detection.

### 2.7. In Silico Analysis

To establish the binding mode of myricetin with different protein targets involved in insulin secretion, we performed molecular docking simulation studies with Epac-2, PKA RIα, and MEK Kinase. In the case of cAMP binding proteins, i.e., Epac-2, and PKA RIα, the binding sites were established using the primary site occupied by cAMP, while for MEK Kinase, the coordinates of the inhibitor UCB1353770 were employed. Crystal structures of Epac-2, PKA RIα, and MEK Kinase were retrieved from RCSB PDB [15] under the respective PDB codes: 3CF6 for Epac-2 [16], 4MX3 for PKA RIα [17], and 3SLS for MEK Kinase [18].

The structures were subjected to protein preparation to add missing residues and atoms and remove chains in the case of multiple chains. The water molecules in the binding site were removed, other than Epac-2 (PDB ID: 3CF6), where water molecules mediating the water bridge were retained. Partial charges were assigned under the Amber99 force field, and hydrogen atoms were added using protonate3D, following a short minimization to optimally orient side chains and hydroxyl moieties. The structure of myricetin was retrieved as smiles from PubChem [19] and was later converted into 3D structures using the Builder module in MOE [20,21]. The compound was later protonated, subjected to charge assignment under MFF94x, and minimized. In all cases, the default placement and scoring methods for the Rigid docking protocol were employed. The top-ranked pose was then analyzed using the Protein-Ligand Interaction Profiler [22,23] web server. For all molecular docking simulation studies, we employed the protocol established earlier [24].

### 2.8. Statistical Analysis

The statistical analyses were conducted using SPSS 27.0 Statistical Package for Windows (SPSS, Inc., Chicago, IL, USA). The values were presented as mean ± S.E.M. ANOVA, followed by post hoc Tukey’s test applied for the comparisons. Statistical significance was determined by *p*-values < 0.05.

## 3. Results

### 3.1. Effect of Myricetin on Insulin Secretion from Isolated Mice Islets

Myricetin (Figure 1A) was evaluated for its insulin secretory effects at basal (3 mM) and stimulatory (16.7 mM) glucose concentrations in a dose-dependent manner. The experiment highlighted that, at basal glucose, myricetin at any dose (1, 5, 25, and 100 µM) did not depict insulin secretion, which indicates that myricetin does not stimulate insulin secretion at the basal glucose level. However, when evaluated at 16.7 mM (stimulatory) glucose concentration, it showed significant insulin secretion in a dose-dependent manner, indicating its potential to stimulate insulin at stimulatory concentrations only (Figure 1B). Myricetin showed significant insulin secretory activity at 5 μM (19.32 ± 1.6 ng/islet/h; *p* < 0.001) and 25 μM (22.01.32 ± 0.7 ng/islet/h; *p* < 0.001) concentrations, when compared with 16.7 mM glucose alone (9.34 ± 1.48 ng/islet/h). No further increase in insulin secretion above 25 μM concentration was observed, suggesting the optimum concentration for further exploration of mechanism(s).

### 3.2. Myricetin Effect Is Glucose-Dependent, Independent of the K_ATP_ Channel

**Figure 2:**
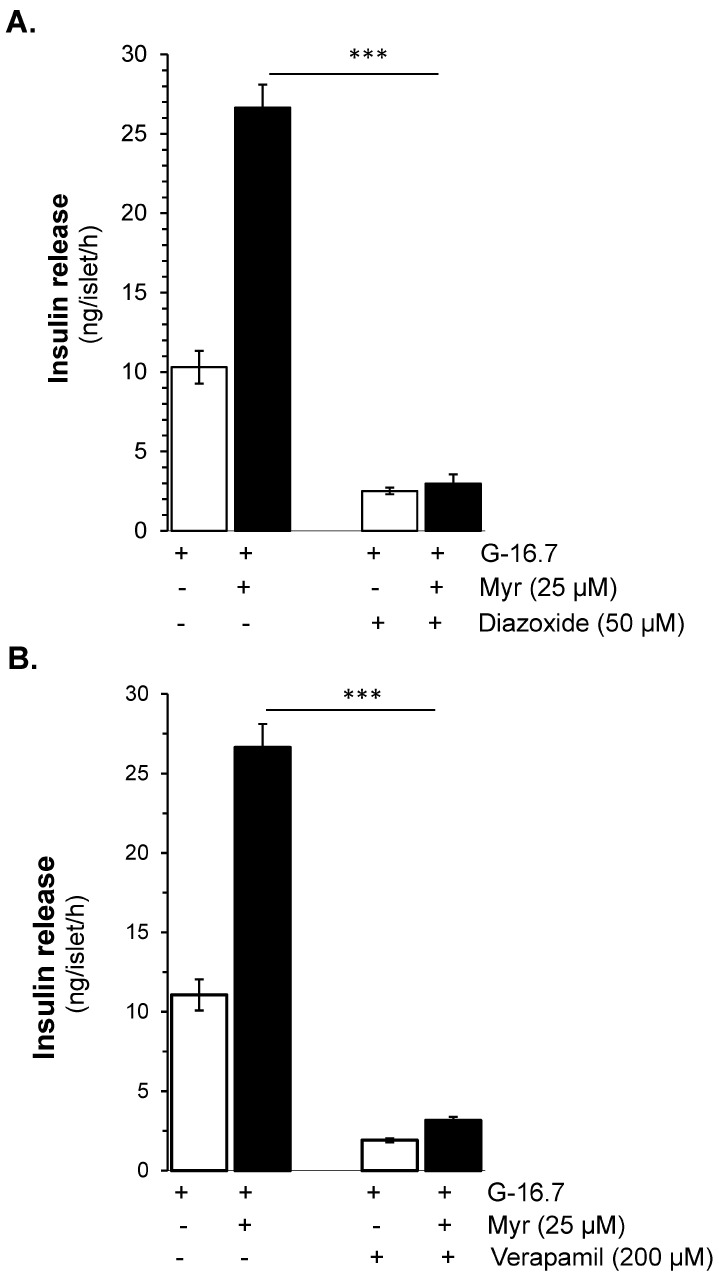
Myricetin enhances glucose-dependent insulinotropic effect independent of K-ATP channels. (**A**) The effect of myricetin on insulin secretion in mice islets with K-ATP channels opened by diazoxide. Mice islets were incubated in 3 mM or 16.7 mM glucose in the presence or absence of myricetin and/or diazoxide (K-ATP channels opener), used at the indicated concentration. (**B**) Effect of myricetin on insulin secretion with Ca^2^⁺ channels blocked by verapamil. Islets were incubated in 3 mM or 16.7 mM glucose in the presence or absence of myricetin and/or verapamil at the indicated concentrations. The mean and SEM for the analysis were calculated based on five independent experiments conducted in triplicates. *** *p* < 0.001, significant changes over the respective control values.

**Figure 3:**
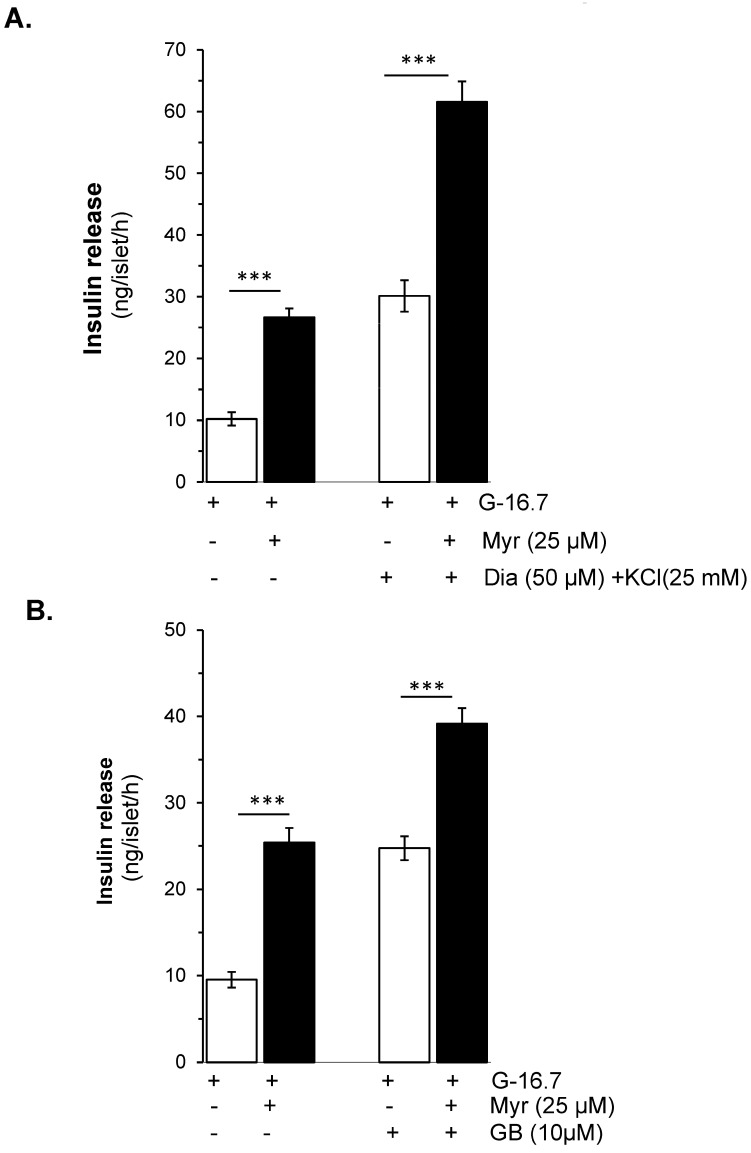
Effects of myricetin on insulin secretion in (**A**), depolarized with Diazoxide- and KCl, and (**B**) Glibenclamide (GB)-treated mice islets. Islets were incubated in 16.7 mM glucose in the presence or absence of myricetin (25 μM) and/or diazoxide (50 μM) + KCl (25 mM) and GB (10 μM). The mean and SEM for the analysis were calculated based on 3 independent experiments conducted in triplicates. *** *p* < 0.001, significant changes over the respective control values.

To evaluate whether the effect of myricetin is glucose- and/or K_ATP_ channel-dependent, the isolated islets were incubated in the presence and absence of diazoxide (K_ATP_ channel opener) with and without myricetin. The insulin secretion potential of myricetin was significantly decreased by diazoxide (2.98 ± 0.6 vs. 26.65 ± 1.5 ng/islet/h) (Figure 2A). Furthermore, to induce membrane depolarization, isolated islets were incubated with KCl (25 mM), and diazoxide (50 μM) to open the K_ATP_ channels, in the presence or absence of myricetin. A significant amplifying effect was observed by myricetin in the depolarized islets in the presence of diazoxide compared to the depolarized islets alone (61.58 ± 3.3 vs. 30.12 ± 12.54 ng/islet/h) (Figure 3A). This exclusive amplifying effect of myricetin was further validated in the glibenclamide-treated islets. In this experimental condition, myricetin augmented the glibenclamide-stimulated insulin secretion further (39.18 ± 1.7 vs. 22.80 ± 1.4 ng/islet/h) (Figure 3B), suggesting the unique insulinotropic potential of myricetin without the direct involvement of K_ATP_ channels, which seems to occur on the intracellular amplifying signaling pathways.

### 3.3. Ca^2+^-Dependent Insulin Secretory Effect of Myricetin

Verapamil (200 μM), a Ca^2^⁺ channel blocker, showed complete inhibition in myricetin-induced insulin secretion at 16.7 mM glucose in isolated islets (3.17 ± 0.3 vs. 26.65 ± 1.5 ng/islet/h, *p* < 0.001). Furthermore, in the absence of extracellular Ca^2^⁺, the myricetin-induced insulin secretion was also completely inhibited (Figure 2B).

### 3.4. Myricetin Role in Inhibition of cAMP Hydrolysis and/or Activation of cAMP Production 

**Figure 4:**
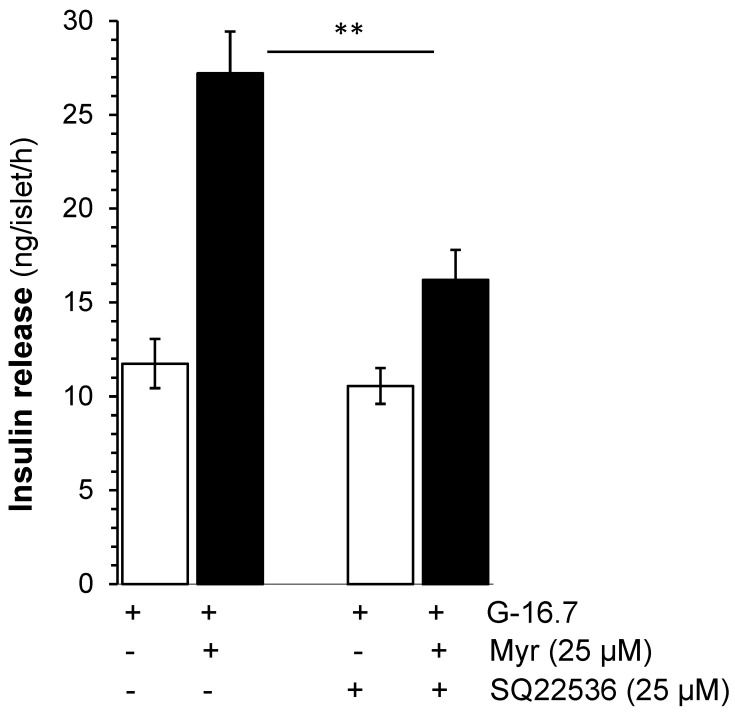
Effects of myricetin on insulin secretion while inhibiting adenylyl cyclase. Islets were incubated in 16.7 mM glucose in the presence or absence of myricetin and/or SQ22536, an adenylyl cyclase inhibitor. The mean and SEM for the analysis were calculated based on 3 independent experiments conducted in triplicates. ** *p* < 0.01, significant changes when compared with myricetin alone.

To evaluate the effect of myricetin on the activation of adenylate cyclase or upstream to the adenylate cyclase, we used SQ22536 (25 µM), which possesses an adenylate cyclase inhibitor effect on the myricetin-induced insulin secretion. The results showed that SQ22536 reduced the myricetin-induced insulin secretion significantly (17.21 ± 1.7 vs. 27.21 ± 2.2 ng/islet/h, *p* < 0.001), but not completely (Figure 4).

**Figure 5:**
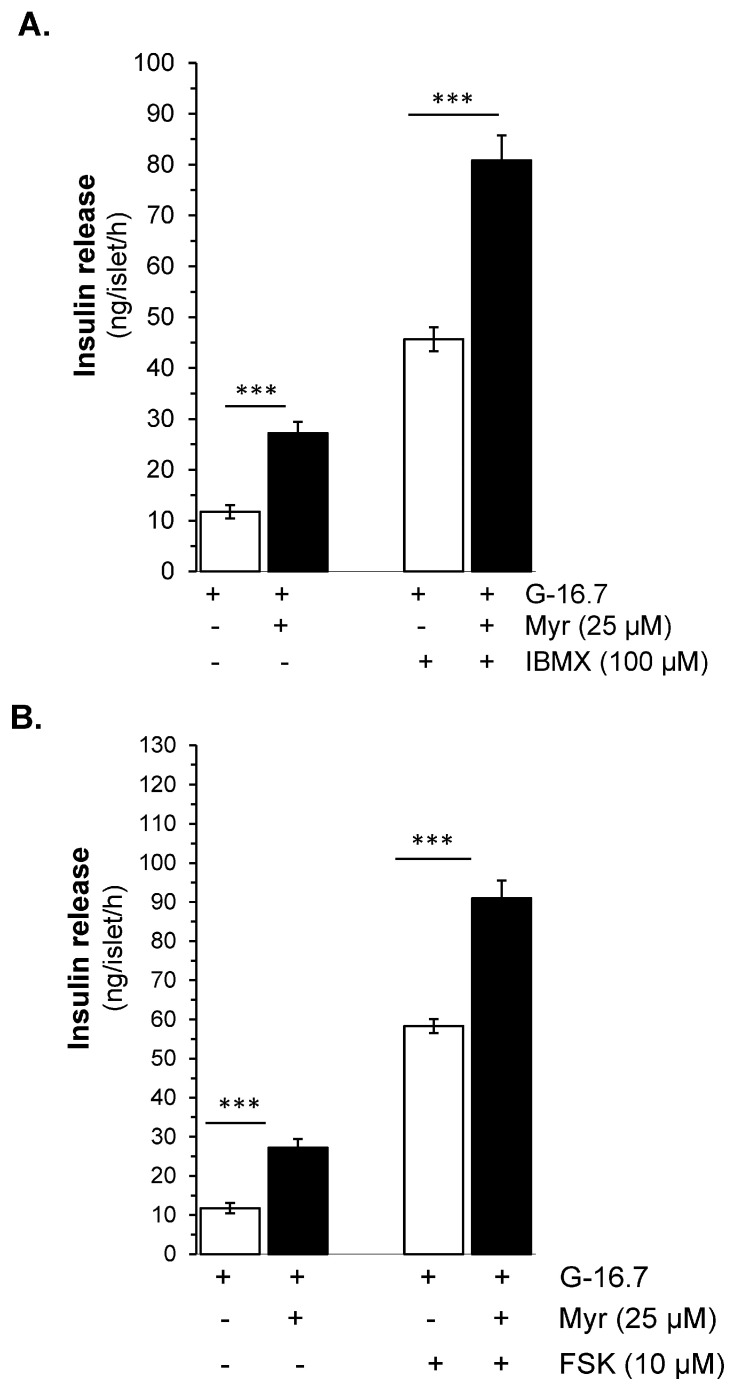
Effect of myricetin on cAMP production and/or inhibition of cAMP hydrolysis. Effect of myricetin on insulin secretion (**A**) in the presence or absence of IBMX, a phosphodiesterase inhibitor, and/or (**B**) FSK, an adenylate cyclase activator. Islets were incubated in 16.7 mM glucose in the presence or absence of myricetin and/or IBMX/FSK at the indicated concentrations. The mean and SEM for the analysis were calculated based on 3 independent experiments conducted in triplicates. *** *p* < 0.001, significant changes when compared with control values.

The inhibition by SQ22536 is significant but incomplete, which led us to investigate myricetin’s effect on downstream signaling pathways. We evaluated whether myricetin affects occur on the inhibition of the production of cAMP by adenylate cyclase activation or the inhibition of the hydrolysis by phosphodiesterase. For both, we used the forskolin (FSK), an adenylate cyclase activator, and IBMX, a phosphodiesterase inhibitor. Interestingly, we found that myricetin further augmented the FSK-induced insulin secretion (91.23 ± 4.5 vs. 59.54 ± 1.7 ng/islet/h, *p* < 0.001), as shown in Figure 5B. Similarly, myricetin further amplifies the IBMX-induced insulin secretion (80.86 ± 4.9 vs. 53.12 ± 2.9 ng/islet/h, *p* < 0.001), as shown in Figure 5A.

### 3.5. PKA Mediated Myricetin-Induced Insulin Secretion

**Figure 6:**
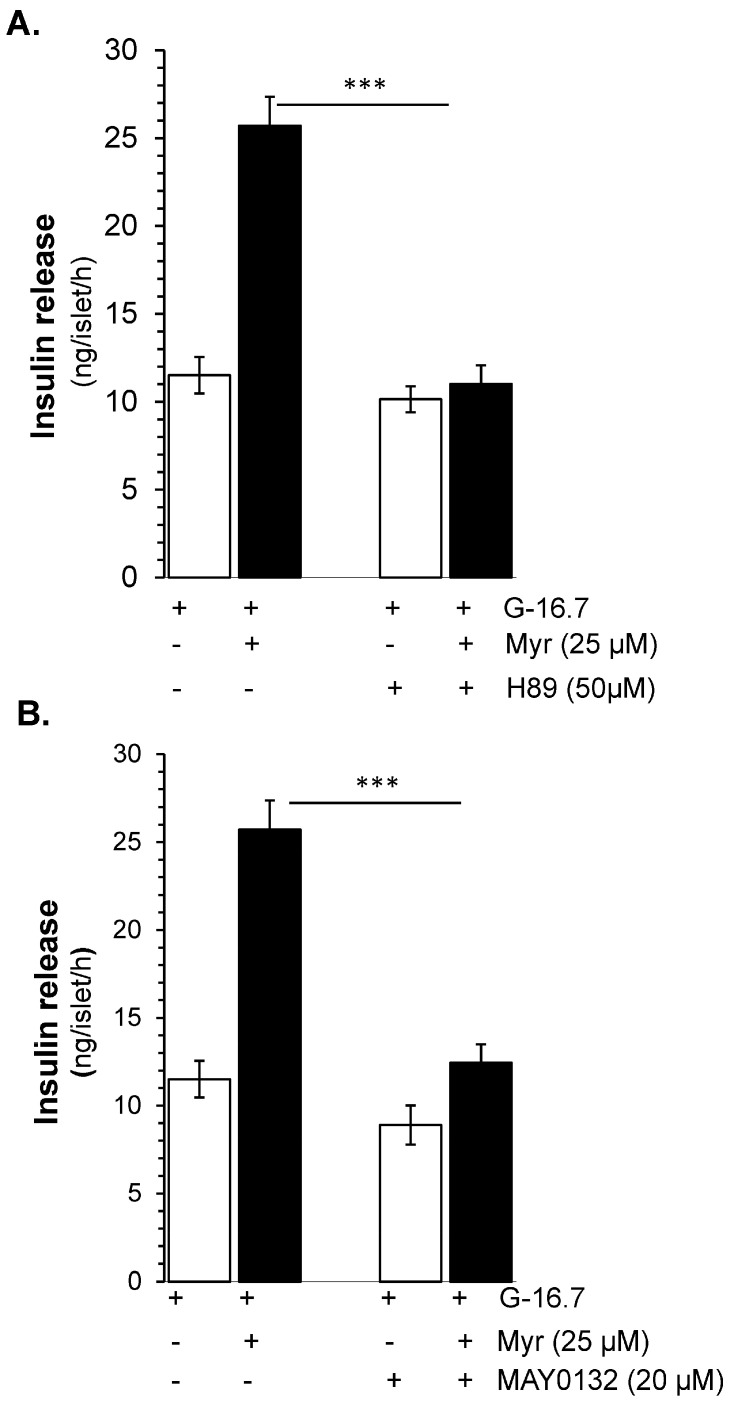
PKA- and Epac2-dependent insulin secretory effect by myricetin. Islets were incubated at 16.7 mM glucose in the presence or absence of myricetin and (**A**) H-89, a PKA inhibitor, and (**B**) MAY 0132 (Epac-2 inhibitor). The mean and SEM for the analysis were calculated based on five independent experiments conducted in triplicates. *** *p* < 0.001, significant changes when compared with myricetin alone.

To evaluate the synergistic downstream insulin secretory effect of FSK and IBMX, on PKA, H-89, a PKA inhibitor, was used. The results showed that H-89 at the dose of 50 µM exhibited significant (almost complete) inhibition of the glucose-stimulated insulin secretory potential of myricetin (11.15 ± 1.06 vs. 25.71 ± 1.7 ng/islet/h, *p* < 0.001), as shown in Figure 6A, which suggests the involvement of PKA in myricetin-induced insulin secretion.

### 3.6. Epac 2 Mediated Insulin Release by Myricetin

Due to the synergistic effect of Epac 2 and PKA [25] we further evaluated the effect of Epac 2 in myricetin-induced insulin secretion. Using the MAY 0132, an Epac-2 inhibitor, we found that the myricetin-induced insulin secretion was almost completely inhibited (12.45 ± 1.71 vs. 25.71 ± 1.7 ng/islet/h, *p* < 0.001) Figure 6B. Interestingly, the pattern of inhibition by Epact 2 is almost similar to the inhibition by PKA.

### 3.7. In Silico Validation of Targets Occupied by Myricetin

**Figure 7:**
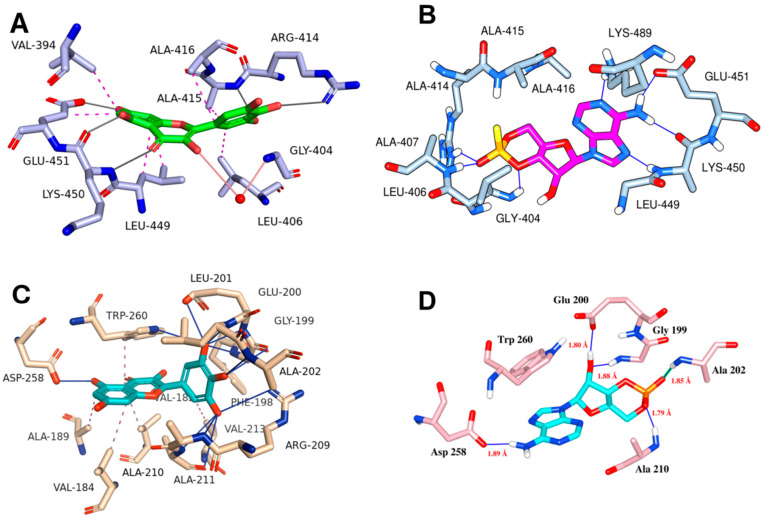
Molecular docking interactions of myricetin with various protein targets. Panel (**A**) illustrates the interaction of myricetin with Epac-2, (**B**) presents the key interactions of cognate ligand cAMP with Epac-2, (**C**) depicts the interaction of myricetin with the PKA RI alpha homodimer (PDB ID: 4MX3), and (**D**) shows the key residue interactions for cognate ligand cAMP of PKA RI alpha homodimer. Hydrogen bonds are indicated as blue solid lines, while hydrophobic interactions are represented as magenta dotted lines, emphasizing the binding interactions and conformational dynamics of myricetin with each respective protein target.

Molecular docking simulations using the ligand myricetin and the Epac-2 protein structure from PDB ID 3CF6 reveal a stable binding conformation supported by a series of hydrophobic interactions, hydrogen bonds, and water bridges. Myricetin adopts a favorable orientation within the Epac-2 binding site, establishing multiple interactions that contribute to its stability (Figure 7A). Myricetin engages in hydrophobic contacts with key residues, including VAL-394, LEU-406, ALA-415, ALA-416, LEU-449, and GLU-451. Specifically, the hydrophobic interactions with VAL-394, LEU-406, ALA-415, and LEU-449 help anchor ligands within the protein’s hydrophobic pocket (Figure 7A). These interactions are crucial for maintaining the structural integrity of the myricetin-Epac-2 complex. Hydrogen bonding interactions further stabilize the complex. Myricetin forms strong hydrogen bonds with ARG-414, ALA-415, LYS-450, and GLU-451. Notably, the hydrogen bond with GLU-451 is particularly stable, indicating a highly favorable interaction. Additionally, a water bridge involving GLY-404 further contributes to the stability of the Myricetin-Epac-2 complex. This water-mediated interaction spans 4.04 Å and plays a supportive role in maintaining the ligand’s position within the binding site.

Similarly, molecular docking simulations of myricetin with the PKA RIα, structure from PDB ID: 4MX3 reveal a stable binding conformation, characterized by a comprehensive network of molecular interaction, including several critical hydrogen bonds within the binding site of the protein, enhancing the stability of the complex (Table 1, Figure 7C). Further, a significant π-stacking interaction is observed between myricetin and the TRP-260 residue. This interaction is characterized by distances ranging from 3.45 Å to 4.42 Å, contributing to the ligand’s binding affinity. The stacking interaction with TRP-260 suggests a stable and planar alignment of myricetin within the binding pocket. Additionally, myricetin also forms a salt bridge with ARG-209. Among other molecular interactions, several critical hydrogen bonding was formed by myricetin with GLY-199, GLU-200, LEU-201, ALA-202, ALA-210, ASP-258, and TRP-260.

**Figure 8:**
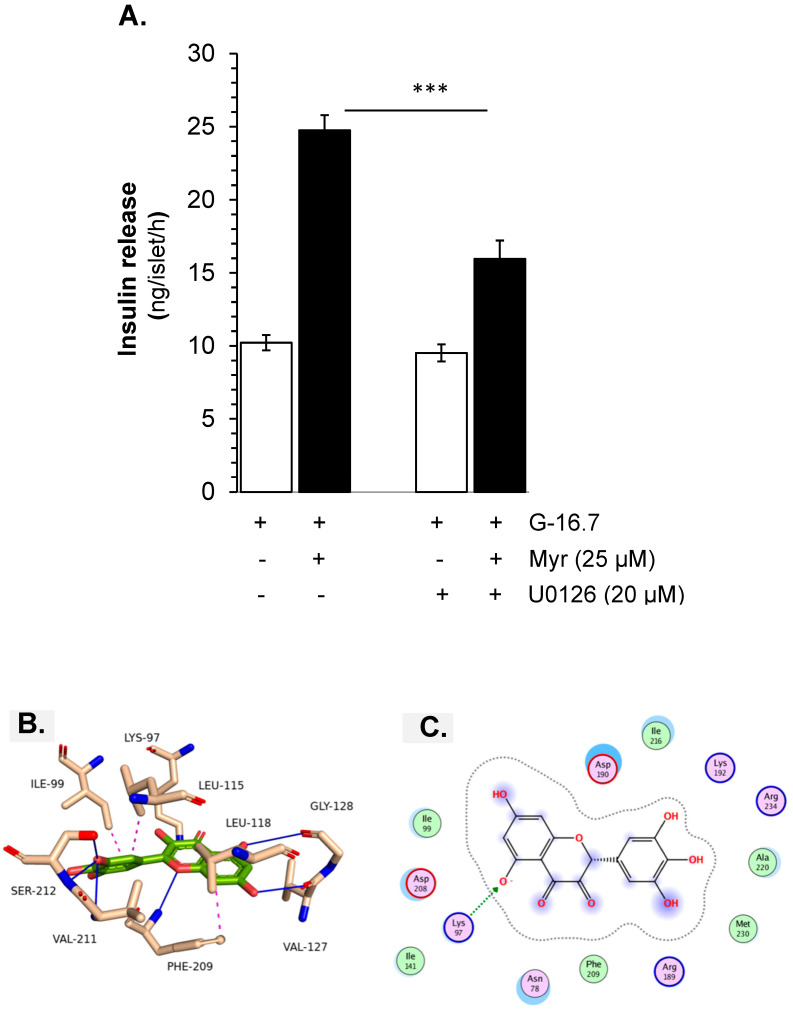
Effect of myricetin on the MEK kinase for the stimulation of insulin secretion. Islets were incubated in 16.7 mM glucose in the presence or absence of myricetin (**A**) U0126, a MEK kinase inhibitor, at the indicated concentrations. The mean and SEM for the analysis were calculated based on three independent experiments conducted in triplicates. *** *p* < 0.001 denotes significant changes when compared with Myr alone. Molecular docking interactions of myricetin with MEK-1 kinase. Panel (**B**) illustrates the interaction of myricetin with MEK kinase, (**C**) presents the 2D interactions of myricetin with MEK-1 kinase.

Molecular docking simulations of the ligand myricetin with the protein structure from MEK-1 kinase reveal a stable binding conformation characterized by a combination of hydrophobic interactions and hydrogen bonds. There are hydrophobic contacts of myricetin with several residues within the binding site, which contribute to the ligand’s stability by embedding myricetin within the hydrophobic core of the protein (Figure 8C). In addition to hydrophobic contacts, myricetin also interacts with LYS-97, VAL-127, GLY-128, PHE-209, VAL-211, and SER-212 via hydrogen bonding (Table 1).

### 3.8. MEK Kinase-Mediated Myricetin-Induced Insulin Secretion

Furthermore, the involvement of PKA and the crosstalk with the MEK kinase signaling pathway, MEK kinase inhibitor U0126, was used. The experimental results showed that U0126 decreased insulin release significantly (16.25 ± 1.2 vs. 24.51 ± 1.2 ng/islet/h, *p* < 0.001), but the inhibition was less, compared to the inhibition by H-89 (Figure 8A–C).

In a nutshell, the binding interactions and overall stability of the complex and the similarity of the bindings of cAMP and myricetin in Epac-2 and PKA, it is anticipated that myricetin recruits both Epac-2 and PKA to induce the observed insulinotropic effects.

## 4. Discussion

Nowadays, the plant-based, new, safe, efficacious, and promising antidiabetic drug candidates are the research priority and have been extensively investigated [6,7,26]. In the continuation of our project, for the identification of natural anti-diabetic drug candidates, myricetin was found as a promising insulin secretagogue with insulin secretory potential at stimulatory glucose. Previous data reported that myricetin’s anti-diabetic effects are attributed to various mechanisms, such as (*i*) inhibiting α-glucosidase and α-amylase to prevent the breakdown of carbohydrates, (*ii*) inhibiting GLUT2 to suppress the absorption of glucose through a specific transporter, (*iii*) increasing GLUT4 expression to improve insulin-dependent glucose uptake into the muscles, (*iv*) acting as an agonist for GLP-1R to enhance insulin secretion, suppress glucagon secretion, and protect pancreatic β-cells, (*v*) DPP-4 inhibitors to prevent GLP-1 degradation, (*vi*) preventing of Human islet amyloid polypeptide (hIAPP) aggregation and NLRP3 inflammasome activation to safeguard pancreatic β-cells, (*vii*) defending against high glucose-induced apoptosis of endothelial cells, (*viii*) defending against diabetic osteoporosis, (*ix*) oxidative stress regulation to prevent DNPs, and (*x*) enhancement of the gut microbiota [27], but to date, the direct insulinotropic effects of myricetin mediated at only stimulated glucose concentration, and the involvement of intracellular pathways was not explored.

Based on the broad antidiabetic potential, its rich traditional history, and most importantly, promising insulin secretory potential through amplifying pathway exclusively at stimulatory glucose, we investigated myricetin’s exclusive glucose-dependent insulinotropic mechanism(s) in our established isolated islets experimental model using pharmacological agonists and antagonists.

The marketed antihyperglycemic drugs, including sulfonylurea and glinides, have been known to target the β-cell K_ATP_ channels for insulin release. However, these drugs, due to their continuous insulin secretory potential irrespective of glucose, lead to hypoglycemia and, if prolonged, cause pancreatic β-cell exhaustion and destruction that further worsens the diabetic condition [28]. Interestingly, myricetin exerts its insulin secretory potential in a dose-dependent manner exclusively at the stimulatory glucose concentration, suggesting a reduction in the hypoglycemia and other adverse effects caused by already marketed drugs like sulfonylurea and glinide. Furthermore, validating the exclusive glucose-dependent and/or K_ATP_ channels-dependent insulinotropic effect, we found that myricetin exerts an amplifying effect in depolarized islets by exclusion of the K_ATP_ channels, suggesting its action is independent of the direct involvement of K_ATP_ channels. Validating these results, we found the additive effect of myricetin in the glibenclamide-induced insulin secretion, depicting a completely different mechanism of myricetin compared to glibenclamide, suggesting its promising behavior compared to these marketed synthetic sulfonylurea drugs. The additive effect of myricetin in both depolarized and glibenclamide-treated islets suggests its effect on the intracellular signaling pathways, independent of the direct influence of the K_ATP_ channels.

The intracellular Ca^2+^ plays an important role in the modulation of signaling pathways, and a change in its concentration equilibrium plays a vital role in insulin exocytosis as well as β cell functionality [29]. Furthermore, when validating the exclusive glucose-dependent and/or Ca^2+^ channel-dependent insulinotropic effect, we found that after excluding the depolarization-dependent Ca^2+^ channels opening using diazoxide, the myricetin-induced insulin secretion was completely inhibited, suggesting that myricetin has no direct effect on Ca^2+^ channels opening. From these results, we may also conclude that myricetin has no direct effect on the release of intracellular Ca^2+^. However, it is important to note that though myricetin has no direct effect on Ca^2+^ channels, based on our experimental evidence, Ca^2+^ is required for the amplification effect of myricetin-induced insulin secretion. First, myricetin exerts an amplifying effect exclusively at the stimulatory glucose concentration; second, myricetin amplifies the insulin secretion in depolarized and glibenclamide-treated islets. Collectively, these experimental evidences suggest the effect of myricetin on pathways, exclusively dependent on stimulatory glucose concentration distal to the K_ATP_ and Ca^2+^ channels.

Furthermore, when investigating the myricetin-induced, glucose-dependent effect through amplifying signaling pathways, we used agonists and antagonists of the key amplifying signaling pathways. Among the insulin secretory amplifying pathways, the cAMP-PKA signaling pathway plays a central role in glucose-mediated insulin release, functioning in tandem with Ca^2^⁺ to regulate insulin secretion [30,31,32]. It is reported that myricetin acts as a GLP-1 receptor activator, hence leading to the insulin secretory effect [30,31,32]. However, in addition to its GLP-1 receptor agonist, our experimental evidence in isolated pancreatic islets showed that myricetin exhibited its dual glucose-dependent potential of the downstream signaling cascade, first inhibiting adenylate cyclase by SQ22536, where a significant but incomplete inhibitory effect in myricetin-induced insulin secretion was observed. Second, myricetin showed an additive effect in forskolin-induced insulin secretion, third, myricetin showed an additive effect in IBMX-induced insulin secretion, fourth, no significant inhibitory effect was observed by inhibiting the phospholipase C (PLC)/Protein Kinase C (PKC) signaling cascade, using U73122.

Based on our experimental evidences, the involvement of the downstream signaling cascade in myricetin-induced insulin secretion was investigated. The downstream PKA signaling cascade is a pivotal mediator of glucose-driven insulin secretion, and importantly, amplifies the process in synergy with Ca^2^⁺ [30,31,32], through both PKA and Epac2-dependent pathways amplify the insulin-mimetic effect in response to the upstream cAMP. [30,31,32]. However, there are claims and counterclaims regarding the cross-talk between PKA and Epac2 signaling cascade, for its downstream effect. Some of the data showed that PKA and Epac2 are activated independently by cAMP, without affecting each other. Some data showed the synergetic effect of both PKA and Epac2, and inhibition of one of these mediators can inhibit the effect of the other [30,31,32]. In addition to the insulin secretory regulation, the cAMP-PKA-Epac2 signaling cascade is considered to be the key to insulin synthesis, pancreatic β-cell regulation, and regeneration. Therefore, this cascade is considered an important drug target for the identification of new therapies. We found that myricetin amplifies the insulin secretion through the downstream cAMP-PKA-Epac2 signaling cascade based on the following experimental observation: first, the myricetin-induced insulin secretion is significantly inhibited by using the PKA inhibitor H89, second, using the Epac2 inhibitor MAY 0132, the myricetin-induced insulin secretion was significantly inhibited and the insulin inhibitory pattern became almost similar to the inhibition by H89, third, the myricetin further amplified the IBMX- and FSK-induced insulin secretion. These results suggest that myricetin amplified the glucose-dependent insulin secretion through the cAMP-PKA-Epac2 signaling cascade, independent of direct modulation of K_ATP_ and Ca^2^⁺ channels. Additionally, when evaluating the cross-talk between PKA and MEK kinases, we found that myricetin-induced inhibition of insulin secretion was significant but not complete. Furthermore, the binding of myricetin with MEK was less optimal compared to PKA and Epac2, confirming the cross-talk but not the full involvement of this signaling cascade. These results highlight myricetin’s promising therapeutic potential as these pathways have pleiotropic effects on insulin synthesis and secretion, pancreatic cell proliferation, and regeneration [33].

Mimicking the *in vitro* findings of myricetin’s potential through cAMP-PKA-Epac-2 pathways, the detailed *in silico* analysis was performed to analyze the affinity of myricetin with the drug targets that were pinpointed through the experimental analysis in isolated islets. The *in silico* analysis of myricetin with the PKA active site residues showed significant hydrophobic and electrostatic interactions, indicating that myricetin primarily functions through PKA, triggering the cAMP-PKA signaling cascade, followed by the activation of the cAMP-PKA-dependent signaling cascade.

**Figure 9:**
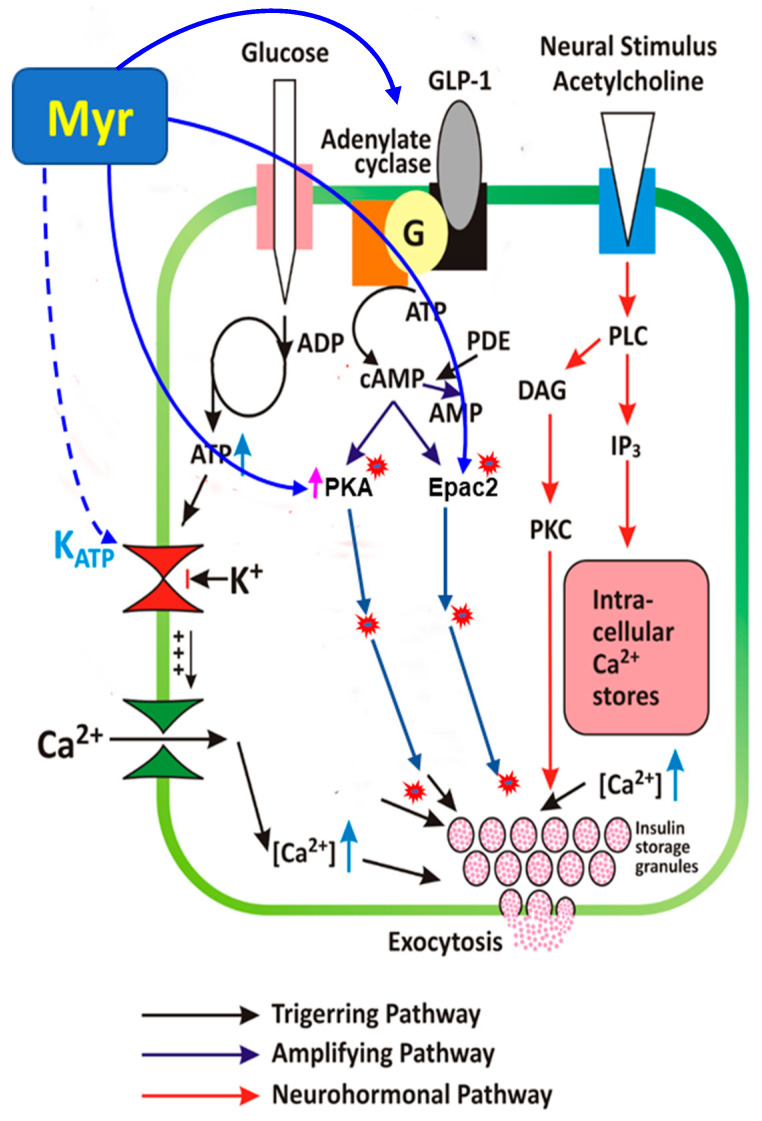
Model of myricetin-induced insulin secretory mechanisms. Myricetin amplifies GSIS in pancreatic islets through modulation of cAMP-PKA-Epac2 signaling cascade, independent of the direct involvement of K_ATP_ and Ca^2+^ channels. Continuous lines ending with arrows head indicate activation; continuous lines ending with block filled head indicate inhibition; dotted lines ending with arrows head indicate indirect involvement. cAMP, cyclic AMP; Epac, exchange protein activated by cAMP; PKA, protein kinase A; PKC, protein kinase C; PDE, phosphodiesterase; DAG, diacylglycerol; IP3, inositol 1,4,5-trisphosphate; RP, reserve pool; RRP, readily releasable pool; K+ -ATP channel, ATP-sensitive K+ channel.

This set of experiments validates that myricetin has an exclusive glucose-dependent insulinotropic effect, which is mediated by the cAMP-PKA-Epac-2 signaling pathway only at stimulatory glucose concentrations. Being a natural compound derived from various plants, myricetin possesses exceptional antidiabetic potential; therefore, it can be a prominent drug candidate for antidiabetic therapy. Furthermore, the interaction of myricetin has been documented with the GLP-1 receptor, and it is hypothesized that by the activation of cAMP, the downregulatory pathways such as PKA and Epac-2 are activated, due to which insulin secretion occurs. However, in the current set of experiments, the effect of myricetin was evaluated only on the downstream insulin secretory pathways; therefore, it is recommended to evaluate the interaction of the GLP-1 receptor and its downstream pathways in the presence of myricetin. Along with the reported data, our experiments also endorse its influence on the insulin regulatory pathways; hence, the compound may be suggested for further evaluation by a controlled clinical trial on type 2 diabetic patients (Figure 9).

## 5. Conclusion

Myricetin, a potential insulin secretagogue, enhances insulin secretion through the cAMP-PKA-Epac2 signaling pathway, but only under high glucose conditions. This glucose-dependent mechanism suggests that myricetin is less likely to cause drug-induced hypoglycemia or other side effects commonly associated with currently marketed antidiabetic drugs. However, further molecular studies are required to precisely identify its drug target.

## Figures and Tables

**Table 1:** Molecular interactions of myricetin with various protein targets, including the types of interactions, interacting residues, and corresponding distances.

Target	Type of Interaction, Residue (Distance)
Epac-2	**Hydrophobic**: VAL-394E (3.96), LEU-406E (3.79), ALA-415E (3.97), ALA-416E (3.80), LEU-449E (3.75), LEU-449E (3.87), GLU-451E (3.58)
	**Hydrogen Bond:** ARG-414E (2.08), ALA-415E (2.15), LYS-450E (1.55), LYS-450E (2.03), GLU-451E (1.59)
	**Water Bridge:** GLY-404E (4.04)
	**Hydrogen Bond:** THR-401C (2.13), VAL-420C (2.20), ASP-481C (2.52)
MEK Kinase	**Hydrophobic**: ILE-99A (3.46), LEU-115A (3.54), LEU-118A (3.45), PHE-209A (3.38)
	**Hydrogen Bond**: LYS-97A (3.02), VAL-127A (2.47), GLY-128A (3.18), PHE-209A (2.38), VAL-211A (2.55), SER-212A (2.83), SER-212A (2.98)
PKA RIα, Homodimer	**Hydrogen Bond:** GLY-199A (1.85), GLU-200A (2.66), GLU-200A (2.95), LEU-201A (2.77), ALA-202A (1.74), ALA-210A (1.91), ASP-258A (2.77), TRP-260A (2.50)
	**π-Stacking**: TRP-260A (4.42), TRP-260A (3.45), TRP-260A (3.90)
	**Salt Bridge**: ARG-209A (4.59)

## Data Availability

The raw data supporting the conclusions of this article will be made available by the authors upon request.
